# Mixed ovarian germ cell tumor in a child: A case report of a rare association

**DOI:** 10.1016/j.amsu.2021.102237

**Published:** 2021-03-29

**Authors:** Larbi Benradi, Kamal El Haissoufi, Abdelouhab Ammor, Youssef Benmoussa, Imane Kamaoui, Anas Haloui, Amal Bennani, Houssain Benhaddou

**Affiliations:** a Uro-visceral and Genital Paediatric Surgery Department, Mohammed VI University Hospital, Oujda, Morocco; b Faculty of Medicine and Pharmacy, Mohammed Ist University, Oujda, Morocco; c Department of Radiology, Mohammed VI University Hospital, Oujda, Morocco; d Department of Pathology, Mohammed VI University Hospital, Oujda, Morocco

**Keywords:** Mixed ovarian germ cell tumor, Surgery, Child, Case report, SCARE

## Abstract

**Introduction and importance:**

ovarian tumors and especially mixed ovarian germ cell tumors are rarely seen in the paediatric population.

**Case presentation:**

we report the case of a 13-year-old girl which was successfully treated for a mixed ovarian germ cell tumor with a favorable evolution.

**Clinical discussion:**

the incidence of mixed ovarian germ cell tumors, clinical manifestations, histologic distribution and prognosis are predominentely distinct in children and adolescents as compared to adult population. The diagnosis should be suspected in young girls with chronic abdominal pain and palpable swelling of the lower abdomen. Conservative surgery is the first therapeutic procedure that consists of a total resection of the mass with preservation of the reproductive function. Circulating tumor markers have the potential in diagnosis, prognostic stratification and for follow-up.

**Conclusion:**

mixed ovarian germ cell tumors are uncommen in children. Their management must be multidisciplinary and conservative surgery by laparotomy represent the standard of care.

## Highlights

• Mixed ovarian germ cell tumors are rarely seen in the paediatric population.
• They must be suspected in young girls with chronic abdominal pain and palpable mass of the lower abdomen.
• Total excision with preservation of ovarian reproductive function followed by chemotherapy is the gold standard of treatment.

## 1 Introduction

Ovarian neoplasms are relatively rare in the paediatric population. Their estimated annual incidence does not exceed 2.2 cases per 100,000 girls and only about one-fourth of all ovarian tumors in females younger than 16 years are described to be malignant (27%) [1]. Moreover, ovarian cancer represents 1.1% of all malignant childhood tumors [2]. Histopathologically and in contrast with ovarian cancer distribution in adults, malignant germ cell tumors (GCTs) are more common than epithelial and sex cord stromal neoplasms [3]. According to the literature, their overall prognosis is reported to be excellent [3]. Despite advences in understanding the etiopathogenesis of malignant ovarian GCTs, their etiology is still not well understood [4]. Here, we report the findings of a mixed ovarian GCT in a 13-year-old girl treated in our primary academic care center. This case report has been reported in line with the SCARE Criteria [5].

## 2 Case presentation

**Fig. 1:**
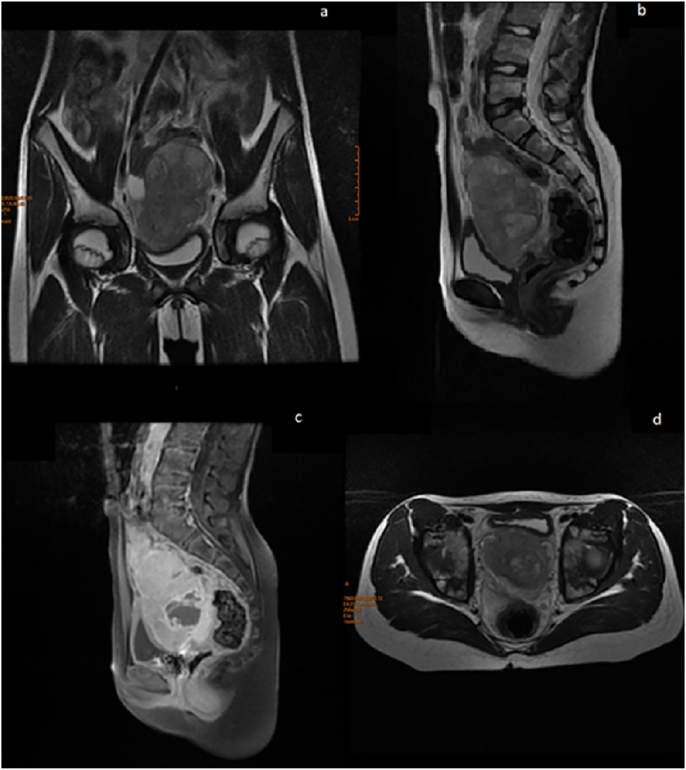
a. Coronal MRI view showing a right ovarian tumor with irregular seams. Peripheral hypersignal and central hyposignal T2 are in relation with a central necrosis**b.** Sagittal and **d.** Transverse MRI views showing bladder compression by the tumor. c. Sagittal MRI view showing a right ovarian tumor on hypersignal T1.

**Fig. 2:**
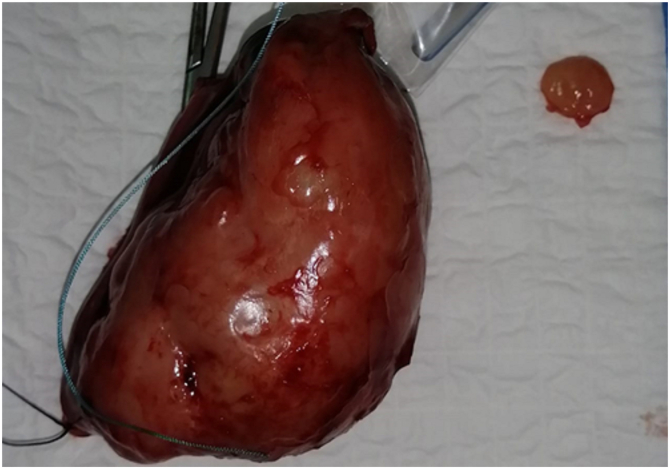
Surgical specimen of right adnexectomy with a peritoneal nodule.

**Fig. 3:**
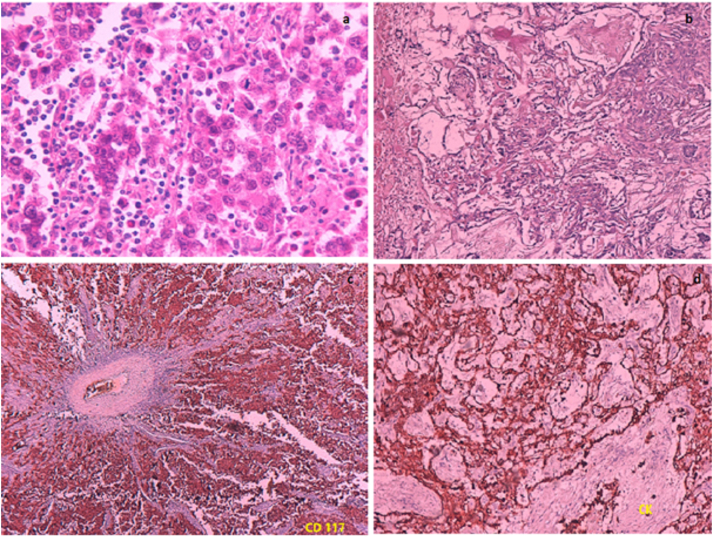
a.The predominant component (dysgerminoma) **b.** The minor component (Yolk sac tumor) **c.** Diffuse cytoplasmic expression of CD117 by tumor cells of the dysgerminoma component **d.** Cytoplasmic expression of Cytokeratine by tumor cells of yolk sac tumor component.

**Fig. 4:**
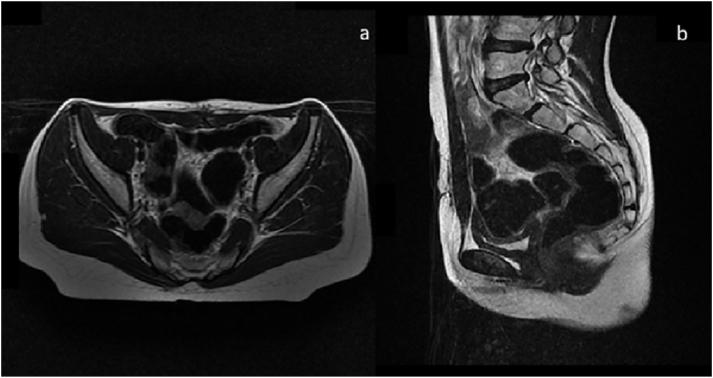
a. transverse and **b.** Sagittal MRI control after 6 months not showing any recurrence or tumor residue.

A 13-year-old girl with no significant pathological history was referred by family physician to our department of Paediatric Surgery for swelling associated with a right abdominal flank pain. The patient had no family, drug history or any past surgery nor a particular genetic predisposition to any diseases. Clinical examination found an apyretic child and in a good general condition. On abdominal examination, a right flank tenderness with a solid mass measuring 12 cm was characterized. Additionally, an abdominal ultrasound was performed and showed a poorly limited right para-uterine mass with 100*52 mm in size. Magnetic resonance imaging (MRI) revealed a right ovarian tumoral mass measuring 97*47 mm with irregular seams, on iso-signal T1, peripheral hypersignal and central hyposignal T2 in relation with a central necrosis (Fig. 1). Moreover, serological alpha-fetoprotein (αFP) level was elevated (490.68 ng/ml) in contrast with a normal level of Beta-human chorionic gonadotropin (β-hCG) (7.77 mU/ml). All other serum parameters were normal. No diagnostic challenges in our patient could be reported. An exploratory laparotomy by a pfannenstiel incision was performed. Intraoperative findings showed a huge mass originating from the right ovary. A right adenexectomy, biopsies of the uterus and a peritoneal fluid sample were carried out (Fig. 2). The whole surgical intervention was performed by a senior paediatric surgeon with the aid of an assistant surgeon and two resident doctors. The postoperative time was uneventful with no hemmoragic or infective complications. The diagnosis of a mixed ovarian germ cell tumor comprising dysgerminoma, as predominant component, and yolk sac tumor as minor component, was made based on the histological appearance, elevated serological level of αFP and immunohistochemical results (Fig. 3). Uterine biopsies and peritoneal fluid sample were found clean from any malignancy. The patient received adjuvant chemotherapy using a combination of vinblastine, bleomycin and cisplatin 21 days apart after the normalisation of αFP in serum. An MRI control was performed and showed no recurrence or tumor residus after six months of follow up (Fig. 4). Also, no negative incidents in terms of adherence and tolerability were observed. Taken together, the evolution of the disease in our patient, our management, and follow-up are summerized in the timeline of Table 1.

**Table 1:** A timeline showing the evolution of the disease in our patient, its medical management and following up.

One year before the consultation	One week before the consultation	Medical consultation	Postsurgical management	After six months of follow-up
Paroxysmal abdominal pain	Worsening of abdominal pain	• Apyrexia and good general condition • Abdominal mass • Abdominal ultrasound and MRI. • Tumor markers • Surgery • Histological diagnosis.	Adjuvant chemotherapy	• No recurrence or tumor residus. • Normalisation of tumor markers.

## 3 Discussion

In children and adolescents, GCTs represent the most common histological type of ovarian tumors and are benign in the majority of cases [6,7]. According to the World Health Organisation (WHO), ovarian GCTs are classified into many histological subtypes including dysgerminoma, yolk sac tumors, embryonal carcinoma, polyembryoma, choriocarcinoma, teratomas and mixed GCTs [8]. The term mixed GCTs is applied to neoplasm containing a combination of malignant germ cell elements. Dysgerminoma followed by yolk sac tumor, which secretes αFP, are the most common subtypes of the ovarian GCTs and particularly concern patients in their second or third decades of life [8]. In our patient, the pathological examination of the resected tumor revealed a combination of these two types.

The clinical presentation of patients with ovarian GCTs can be similar in both benign and malignant forms; the most common signs are the presence of a mass of the lower abdomen and abdominal pain which is usually chronic [9]. This neoplasm is generally asymptomatic or minimally symptomatic as long as it does not reach a considerable size and without compression of the adjacent organs. Vaginal bleeding, constipation or amenorrhea can also be revelating signs but less frequently. In other cases, a complication such as the rupture of the mass, infraction or a torsion may reveal the disease [9].

Dysgerminomas appear usually as a mass containing multiple lobules that are divided by fibrovascular intensively enhanced septa on MRI and computed tomography (CT) imaging [10]. These septa can be shown on Doppler ultrasound with an intensive flow signal [11]. In addition, the neoplasm can contain calcifications, hemorrhagic foci or necrotic areas [12]. As tumor markers, β -hCG levels are rarely high and Lactic Acid Dehydrogenase (LDH) levels are more reliable for diagnosis and follow-up [[13], [14], [15]].

Radiologically, a yolk sac tumor has the property to appear as a voluminous mass with heterogeneous composition: cystic and solid components with areas of necrosis and hemorrhage [16]. The MRI appearance of the tumor can reveal predominency of the solid component contrasting with some areas of cysts and hemorrage, and hypervascular properties through striking contrast enhancement and multiple signal voids. The tumor releases αFP in serum and its levels can be used for diagnostic and follow-up after surgery [17].

The initial surgical procedure is crucial as it is the first therapeutic option consisting mainly of ovariectomy or ovarosalpingectomy which allow the diagnosis of the disease as well as its extension. Depending on the malignant behavior of the neoplasm and the tumor size, surgery can be performed by a transverse infraumbilical or a Pfannenstiel incision or through a midline approach. During the operation, the following staging priciples should be considered according to the Children's Oncology Group for pediatric ovarian germ cell neoplasms:

1. Intact ovarian removal without rupture of the tumor capsule. A salpingectomy must be performed if the fallopian tube is adherent.
2. Examination of the contralateral ovary with biopsy if a suspicious aspect is seen.
3. Inspection of the peritoneum, the liver and the omentum and resection of any abnormal tissue.
4. Inspection of aorto-caval and iliac lymph nodes and biopsy of suspicious ones.
5. Sampling of ascitic fluid for cytological examination. If ascites is absent, a washing is required [18,19].

Biopsy by laparoscopic approach is the best option in case of involvement of neighboring structures or if there is an evidence of a bilateral form. When the initial surgical approach consists of a biopsy or an excision with micro/macroscopic residues or in patients with metastatic disease, a neoadjuvant chemotherapy is recommanded. A post-chemotherapy evaluation show generally a surgically resecable tumor. If the tumor is bilateral, the majority of authors recommend ovarian preservation on the least involved side. Mutilating excisions and bilateral ovariectomies are accepted only when chemotherapy is not effective [20]. Despite the marked favorable outcomes after our management of this patient, our case reporting is limited by the single institution experience. Additional survival studies with prospective enrollment are awaited to confirm these findings.

## 4 Conclusion

Ovarian tumors and especially mixed ovarian germ cell tumors are uncommen in children. Their management must be multidisciplinary between pediatricians, pediatric surgeons and pathologists. Surgery is the standard of care, which should be conservative to preserve reproductive function. A laparotomy approach still the decision of choice if malignancy is suspected, surgical staging is required, and in case of large tumors.

During her last visit and follow-up, our patient achieved a complete remission and her family was satisfied with our management strategy.

## Provenance and peer review

Not commissioned, externally peer-reviewed.

## Sources of funding

None.

## Ethical approval

Not required.

## Consent of patient

Written informed consent was obtained from the patient for publication of this case report and accompanying images. A copy of the written consent is available for review by the Editor-in-Chief of this journal on request.

## Author contribution

Larbi Benradi collected patient's data. Larbi Benradi and Kamal El Haissoufi conducted the literature review, and wrote the manuscript. Abdelouhab Ammor, Youssef Benmoussa, Imane Kamaoui, Anas Haloui, Amal Bennani and Houssain Benhaddou reviewed and supervised the case report writing. All authors approved the final version of this paper.

## Research registration

Not applicable.

## Guarantor

Houssain Houssain and Larbi Benradi.

## Declaration of competing interest

The authors have no conflict of interest to declare.
